# Human primary endothelial label-free biochip assay reveals unpredicted functions of plasma serine proteases

**DOI:** 10.1038/s41598-020-60158-4

**Published:** 2020-02-24

**Authors:** Márta Lídia Debreczeni, Inna Szekacs, Boglarka Kovacs, Andras Saftics, Sándor Kurunczi, Péter Gál, József Dobó, László Cervenak, Robert Horvath

**Affiliations:** 1 3rd Department of Internal Medicine, Semmelweis University, Budapest, Hungary; 2 Nanobiosensorics Momentum Group, Institute of Technical Physics and Materials Science, Centre for Energy Research, Konkoly-Thege M. út 29-33, H-1120 Budapest, Hungary; 3 Institute of Enzymology, Research Centre for Natural Sciences, H-1113 Budapest, Hungary

**Keywords:** Nanoscale biophysics, Imaging and sensing

## Abstract

Tissue-on-a-chip technologies are more and more important in the investigation of cellular function and in the development of novel drugs by allowing the direct screening of substances on human cells. Constituting the inner lining of vessel walls, endothelial cells are the key players in various physiological processes, moreover, they are the first to be exposed to most drugs currently used. However, to date, there is still no appropriate technology for the label-free, real-time and high-throughput monitoring of endothelial function. To this end, we developed an optical biosensor-based endothelial label-free biochip (EnLaB) assay that meets all the above requirements. Using our EnLaB platform, we screened a set of plasma serine proteases as possible endothelial cell activators, and first identified the endothelial cell activating function of three important serine proteases – namely kallikrein, C1r and mannan-binding lectin-associated serine-protease 2 (MASP-2) – and verified these results in well-established functional assays. EnLaB proved to be an effective tool for revealing novel cellular mechanisms as well as for the high-throughput screening of various compounds on endothelial cells.

## Introduction

There is an ever-increasing demand to develop reliable biological model systems to study cellular function or to test the effect of drug candidates and other bioactive compounds. Modern technology opened up the possibility to deposit living cells or even reliable tissue models directly on a transducer surface to monitor their behavior in a miniaturized, fast and cost-effective manner. These lab-on-a-chip systems are more and more popular in applied biotechnology and their application in basic cell biological research is on the rise^1^. Moreover, such robust and portable devices with primary cells or patient tissues lay the foundation for point-of-care testing in medical diagnostics^2^.

The cells in our body are highly complex machines, which are required to react immediately and adequately to the molecules of their environment in order to maintain homeostasis. Although there are several conventional methods suitable for studying cell behavior, these are often low-throughput and time-consuming assays, and usually rely on the detection of labeled molecules, which can *per se* affect cellular functions, as they may activate unforeseen signaling pathways.

To overcome this problem, the introduction of non-invasive label-free technologies is necessary. Real-time and high-throughput biosensor technologies as the basis of new-generation cell-based screening techniques provide analytical information by the recognition of biological events employing a physical transducer. Resonant waveguide grating (RWG) based optical biosensor technologies utilize an evanescent electromagnetic field to *in situ* monitor refractive index changes close to the surface of the sensor in 96- or 384-well microplates. The detection is typically limited to a probing depth of ~150 nm due to the exponentially decaying electromagnetic evanescent field of the sensing waveguide mode^3,4^. The technology is commercially available and was successfully employed previously to monitor the kinetics of cell–surface and cell membrane receptor–ligand interactions^3,5–7^, binding affinity^8^, cellular signaling^9,10^, cytotoxicity^11^, nanoparticles^12^ and the functional state of surface-adhered cells down to the single cell level^13^. The output of the sensor is an integrated signal of dynamic mass redistribution (DMR) taking place in the sensing depth above the bottom of each well^14^. The Epic BT platform is particularly suitable for the primary screening of the cellular effects of yet uninvestigated substances, as almost any changes in cellular function can affect the refractive index monitored at the bottom surface of the adhered living cells. Therefore, valuable information about nanometer-scale cellular and molecular changes can be easily obtained in real-time and completely label-free manner providing an outstanding possibility far from being fully explored in basic and applied cell-biological researches.

As constituents of the inner lining of all vessel walls, endothelial cells (ECs) are among the first to come into contact with the components of the blood. Numerous important physiological processes are regulated by ECs, for example, vascular permeability, which is controlled by the alteration of the tightness of inter-endothelial adhesion junctions^15^. As ECs are present everywhere in the body in great numbers and are affected by the pathomechanism of several diseases (e.g. atherosclerosis, edematous diseases, sepsis, cancers, etc.), it is particularly important to understand how activated blood components and intravenously administered pharmacological agents affect EC behavior.

Among blood components, enzymes (mostly serine proteases) of hemostasis and the innate immune response are indispensable for maintaining the integrity of the body. Blood serine proteases constitute a very complex and highly interconnected network, which – although only for didactic reasons – is divided into three main cascades, namely the coagulation/fibrinolytic, the kinin-kallikrein, and the complement systems. Coagulation and fibrinolysis are two extremely interlocking processes that are required to ensure the integrity of blood vessels by the occlusion and reparation of injured vessels while keeping the vast majority of blood plasma in a fluid state. The kinin-kallikrein system is the main source of the proinflammatory mediator bradykinin, a small molecule with diverse physiological roles. The complement system is the most important humoral arm of innate immunity that can effectively recognize and eliminate most pathogens immediately after entering bodily fluids^16^. Some plasma serine proteases – for example thrombin and mannan-binding lectin-associated serine protease-1 (MASP-1) of the coagulation and complement cascades, respectively – are known to directly affect EC behavior by the cleavage of cell surface protease activated receptors (PARs)^17^, but most plasma serine proteases have not yet been tested on ECs.

Human umbilical vein endothelial cell (HUVEC) culture is a widely accepted *in vitro* model of the inner vessel walls. Gelatin coating is mainly used to assist HUVECs adherence, however, its application on the biosensor surface should be carefully considered. The challenge is to create a film of gelatin on the biosensor surface thin enough to sense cellular DMR in the exponentially decaying evanescent optical field, while still providing an ideal and robust matrix for EC attachment and growth. The thickness and stability of gelatin were characterized using quartz crystal microbalance (QCM), optical waveguide lightmode spectroscopy (OWLS), atomic force microscopy (AFM) and RWG techniques.

OWLS is a powerful waveguide-based biosensor technique that enables the simultaneous determination of the surface mass, refractive index, thickness and internal ordering of self-assembling thin films^18–22^. Note, the determined optical thickness is usually underestimated in case of heavily hydrated layers^19,22^. While OWLS measures the mass of polymer chains only (“dry” mass), the mechanical biosensor QCM is sensitive both to the polymer chains and to the associated solvent molecules, and measures the so-called cumulative “wet” or hydrated mass as well as the corresponding hydrated thickness^23^. Importantly, the combination of OWLS and QCM data enables to determine the mass of bound water and the hydration degree of hydrogel-based coatings^22,24^. In addition, QCM is also sensitive to the rigidity of the formed layer and quantitative viscoelastic data, such as viscosity and shear elastic modulus, can also be obtained^22,25–27^.

**Figure 1:**
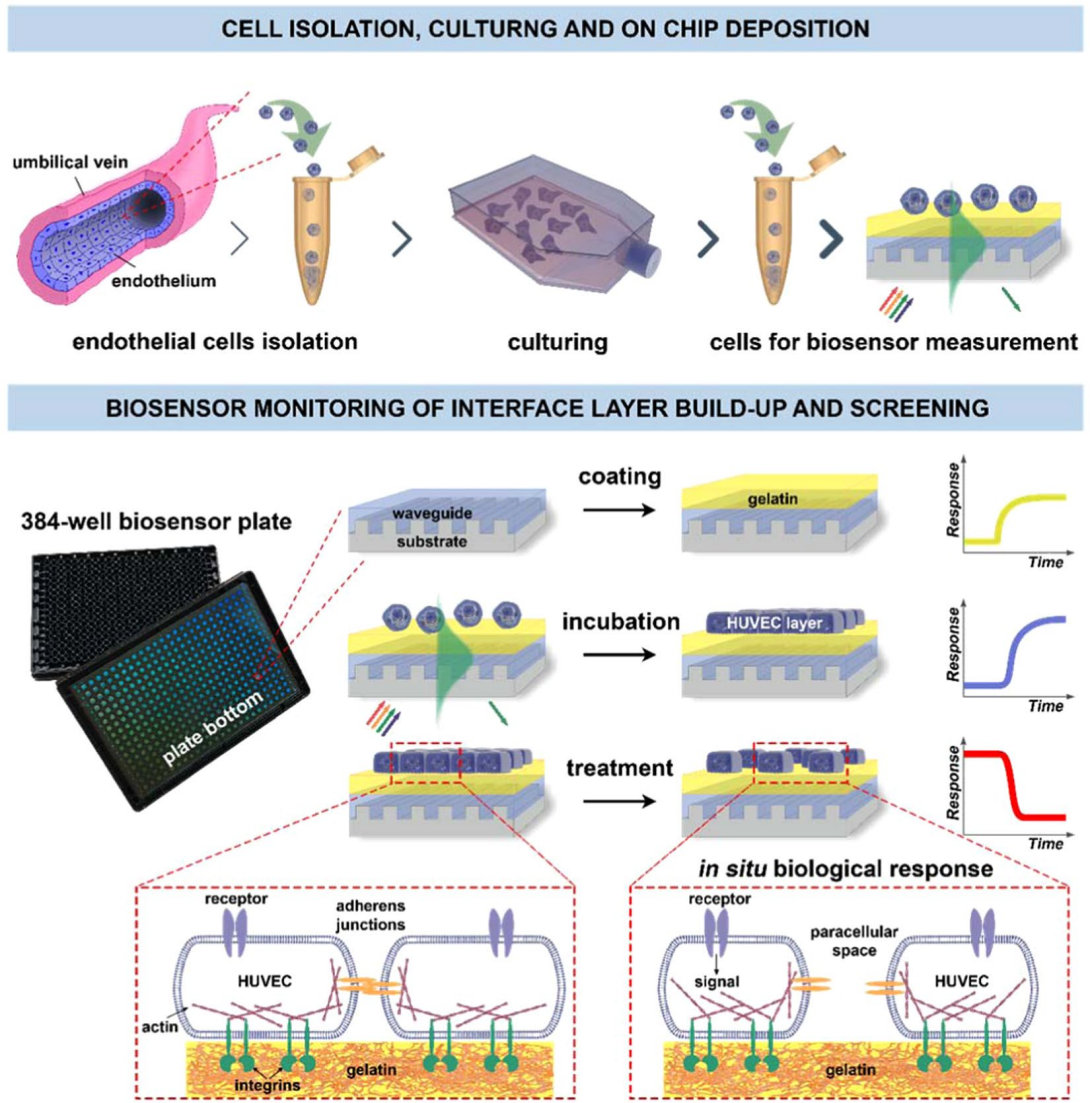
Schematic overview of the proposed EnLaB measurement setup. Upper part: cell preparation steps (primary cell isolation, culturing, transferring onto the sensor chip surface). The incoupled waveguide mode intensity profile is shown in green color. The exponentially decaying evanescent field of the mode senses the adhering cells. The waveguide mode is excited by illuminating the grating structure from below using a broad range of wavelengths. The resonant wavelength excites the waveguide mode which – after propagating a short distance inside the waveguide – couples out into the direction of the substrate (shown in green). The lower part illustrates the steps of the biosensor measurements and typically obtained biosensor responses (the detected shifts in the resonant wavelength): coating of the chip with gelatin, following with cell attachment to the gelatin surface, and subsequent cell treatment by the studied molecular compounds (screening). Illustration of the biological effect of the treatment is highlighted in dashed boxes.

For the screening of plasma serine proteases on ECs, we first demonstrate a label-free, high-throughput and easy-to-use tissue on a chip system, which is perfectly suitable for studying complex cell behavior on an optimized surface coating. In our work, the recently developed resonant waveguide grating based technology was combined with human primary endothelial cell layers in an elegant way (endothelial label-free biochip (EnLaB), see Fig. 1). Using EnLaB, we are the first to describe a novel function of plasma serine proteases, i.e. MASP-2, kallikrein and C1r were able to activate the ECs, which function was validated in various traditional assays.

## Results

### Biosensor surface modification: optimization of gelatin coatings for cell seeding

Since *in vitro* culturing of HUVECs requires an appropriate surface, initial experiments were conducted to optimize the sensor surface coating protocol to achieve reproducible and stable measurement conditions. However, being primary cells, HUVECs are not as easily accessible, therefore “more valuable” than immortalized cell lines, such as HeLa cells, which are also adherent, but much less vulnerable cells. For this practical reason, HeLa cells were used in these initial, surface optimization experiments.

**Figure 2:**
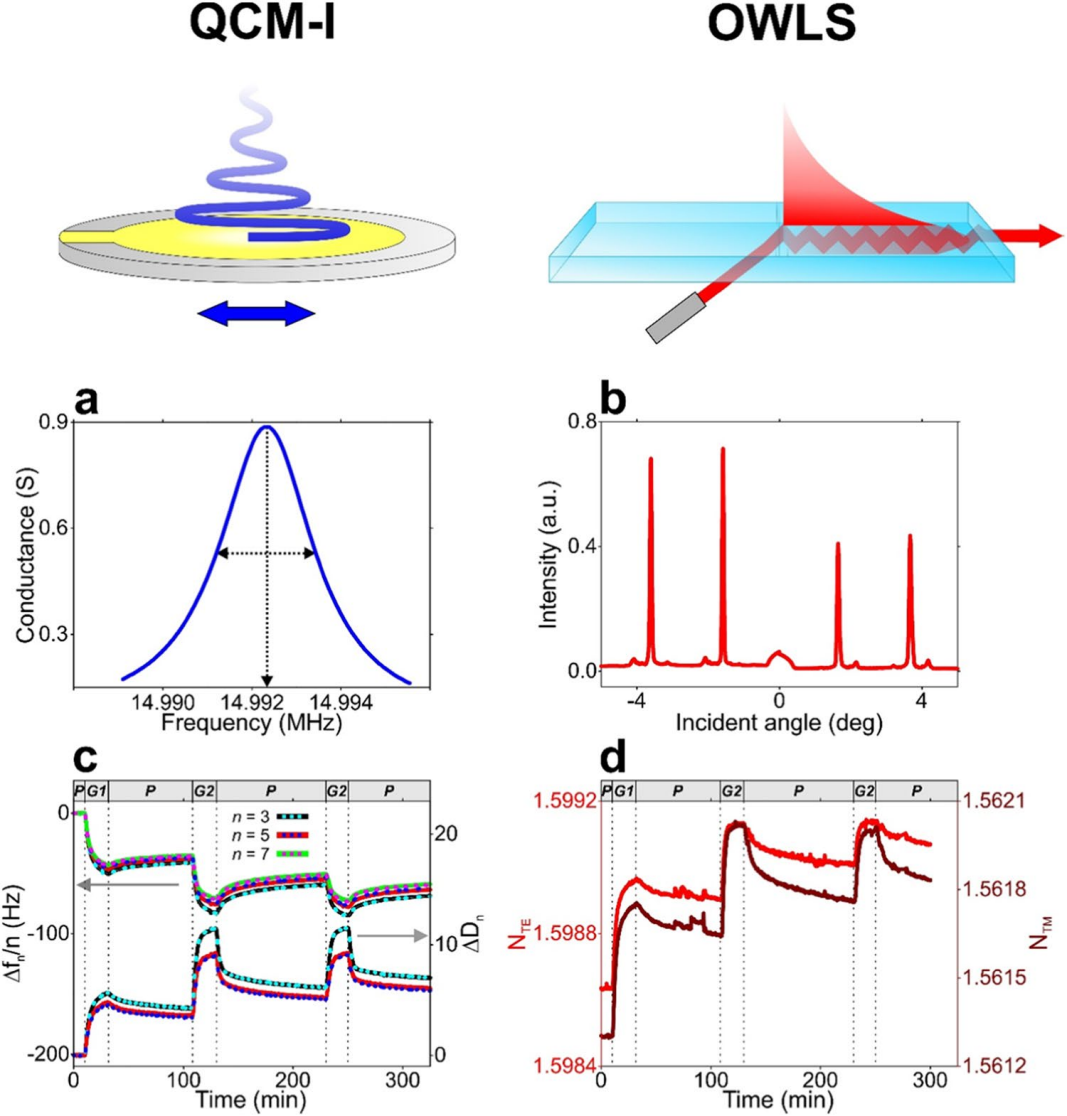
Measurement principle of the QCM-I and OWLS biosensor techniques and obtained raw data from *in situ* measurements on gelatin layer formation. (**a**,**b**) Raw data obtained from QCM-I and OWLS the measurements: the resonance peak around the 3^rd^ overtone resonance frequency (**a** QCM-I) as well as the intensity peaks of the excited modes at the coupling angles (**b** OWLS). In the case of QCM-I, the frequency and full width at half maximum (FWHM) value of the resonance peak are registered at each measurement time and the resulting normalized frequency shift (Δ*f*_*n*_/*n*) and dissipation shift (Δ*D*_*n*_) data (the latter calculated from FWHM) at the selected *n* overtones are used for the model fit. (**c**) Gelatin deposition experiment monitored by *in situ* QCM-I. The measured Δ*f*_*n*_/*n* and Δ*D*_*n*_ data are represented by solid lines with different colors, while the data resulting from fitting the Voigt-based viscoelastic model are represented by colored dashed lines. The graph header and dashed vertical lines indicate the phases of the deposition experiment (P: PBS flow, G1: flow of 0.2 mg/ml gelatin solution, G2: flow of 2 mg/ml gelatin solution). (**d**) Effective refractive index data corresponding to the transverse electric (*N*_TE_, red line) and magnetic modes (*N*_TM_, brown line) obtained by parallel OWLS measurement on gelatin layer formation (the experiment was performed with the same phases detailed above for QCM-I).

**Figure 3:**
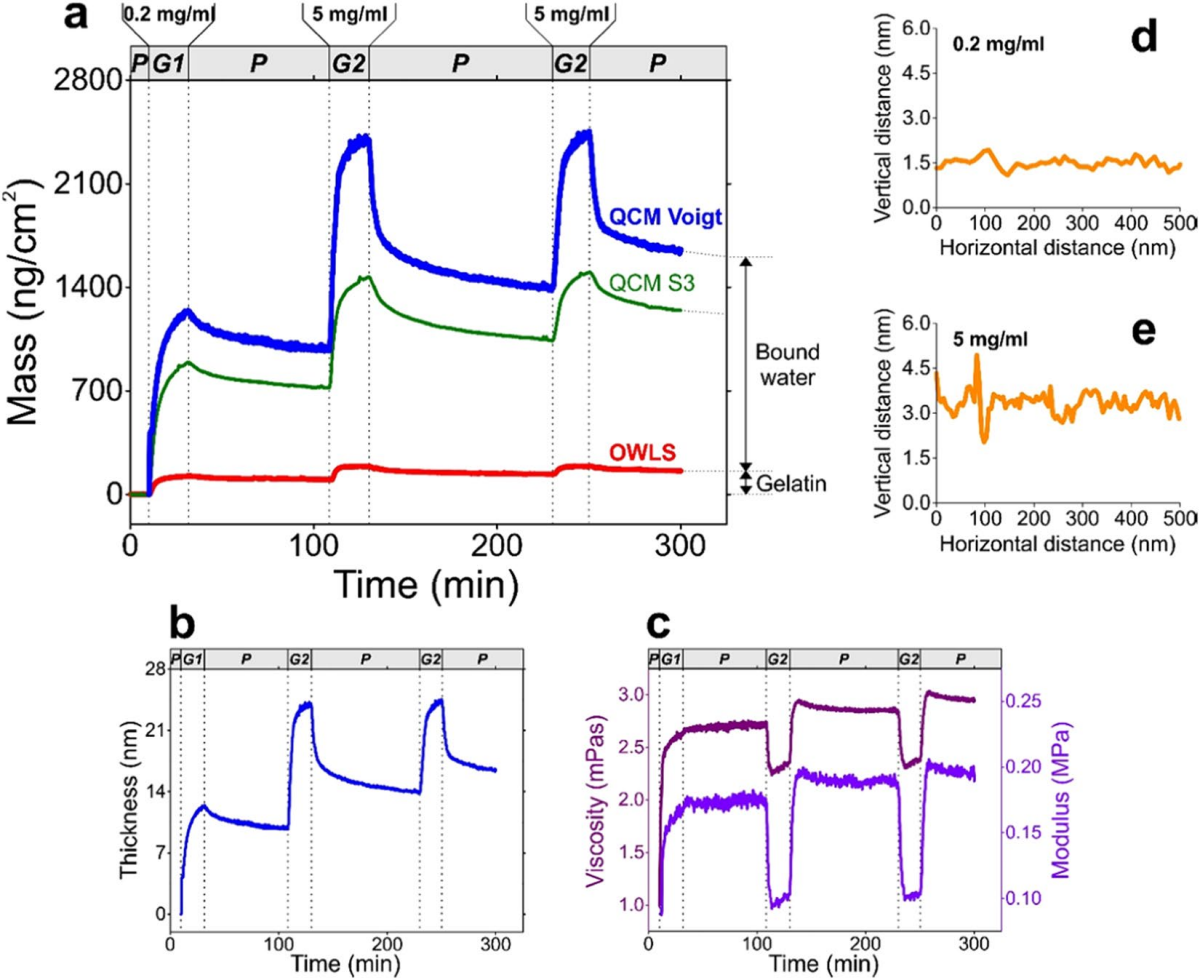
Results obtained from the combined evaluation of QCM-I and OWLS measurement data, supplemented by AFM measurements. (**a**) Areal mass density of the formed gelatin layer, resulting from QCM-I and OWLS measurements. The graph header and dashed vertical lines indicate the phases of the experiment (P: PBS flow, G1: flow of 0.2 mg/ml gelatin solution, G2: flow of 2 mg/ml gelatin solution). While the mass curve derived from the OWLS measurement (red line) represents the mass of the gelatin chains itself (“dry” mass), the mass data obtained from QCM-I represent the mass of the hydrated gelatin layer (“wet” mass). Two types of hydrated mass data were calculated: one relying on the Sauerbrey equation (thin green line, calculated from the 3^rd^ overtone frequency shift data) as well as the other one resulting from the fit of the Voigt-based viscoelastic model (thick blue line). The difference between the “wet” and “dry” masses indicates the amount of bound water. (**b,c**) Thickness (**b**) and viscoelastic properties (**c**) of the formed gelatin layer (shear viscosity (purple line) and shear elastic modulus (red line)), calculated by fitting the Voigt-model. The experimental phases are indicated as above in graph (**a**). The thickness is proportional to the mass data of graph (**a**) with the factor of layer viscosity, estimated to be 1000 kg/m^3^. (**d,e**) AFM line scans of gelatin surfaces prepared from 0.2 mg/ml (**d**) and 5 mg/ml (**e**) gelatin solutions.

Figure 2 presents the scheme of the measurement principle of QCM-I and OWLS as well the curves of the graphs of the raw data. The results on the formation of gelatin coatings obtained by *in situ* QCM-I and OWLS as well as AFM measurements are summarized in Fig. 3. While the QCM-I measurement relies on a generated standing acoustic plane wave, OWLS employs the evanescent wave that is generated by the excited waveguide modes. The primary data are the resonant frequency as well as the coupling angle corresponding to the excited acoustic overtones and waveguide modes, respectively (Fig. 2a,b). From these data, normalized resonant frequency shift (Δ*f*_*n*_*/n*) and dissipation shift (Δ*D*_*n*_) values as well as effective refractive index values (*N*_TE_, *N*_TM_) are subsequently calculated (Fig. 2c,d)^22^.

The combined analysis of QCM-I and OWLS data revealed the hydration and viscoelastic properties of the gelatin coating. Based on the OWLS and QCM-I mass data evaluation, it was found that the hydration degree of the layer prepared using the 5 mg/ml solution is 90% as shown in Fig. 3a (hydration degree is defind by the surface mass of bound water relative to the hydrated mass). When the 0.2 mg/ml solution was used, the hydrated thickness of the gelatin layer was 9.8 nm, which increased to 16.1 nm when the 5 mg/ml solution was subsequently employed (see Fig. 3b). The obtained shear viscosity (*η*) and shear elastic modulus (*μ*) data (Fig. 3c) are in good agreement with literature values measured for hydrogel-type agarose layers (according to the publication of Dutta *et al*., *η* = 2.7 mPa·s and *μ* = 0.21 MPa were obtained for agarose gels)^28^.

The AFM images showed a homogeneous gelatin coating with both gelatin concentrations on the surface, which were very smooth with typical RMS values of 0.2–0.4 nm on a standard 1×1 µm surface, ideal for optical biosensor measurements. Note, however, that more particles were observed on the 5 mg/ml coated samples. The particles are presumably formed from the gelatin by aggregation, the size of which ranged from 20 nm to 200 nm for the 5 mg/ml samples and which were generally smaller for the 0.2 mg/ml samples (Fig. 3d,e).

**Figure 4:**
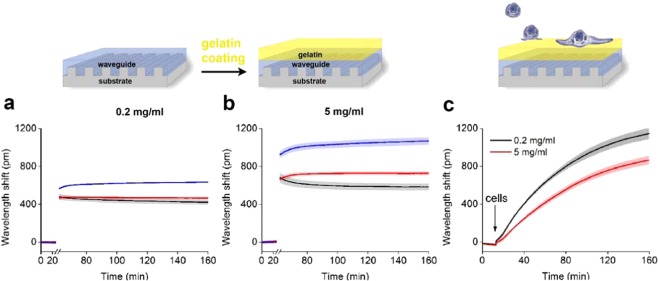
Characterization of sensor coating**s** by the Epic BT. (**a**,**b**) Sensor responses after the coating with gelatin (**a** 0.2 mg/ml. **b** 5 mg/ml). Breaks represent 30 min incubation time outside the instrument. Black: washing two times with PBS, Red: replacement of the solution with PBS, Blue: no further treatment. (**c**) Typical cell adhesion curves obtained on gelatin (0.2 mg/ml, 5 mg/ml) coatings after the washing step. At 15 min, 20,000 HeLa cells were pipetted into the wells of the sensor plate in 20 μl HEPES HBSS buffer, and recorded for 145 min.

The coating procedure was finally tested with Epic BT biosensor plates. After recording the baseline signal, the gelatin coating was performed at 37 °C, outside the Epic BT instrument. Then, the biosensor plate was placed back to the instrument to assess the magnitude and stability of the coating signal. The wavelength shift with 0.2 mg/ml gelatin showed stable signal in two cases, when the washing step was applied (Fig. 4a, black line), and when the gelatin solution was replaced with PBS (Fig. 4a, red line). In contrast, when the gelatin solution remained in the well (Fig. 4a,b, blue line), a concentration-dependent drift was observed. A slight drift was also observed in the case of the 5 mg/ml solution, when it was only replaced with PBS (Fig. 4b, red line). These data show that the critical step of coating the surface with gelatin is the removing of its excess from the surface. It is noteworthy that the higher concentration of gelatin resulted in a larger biosensor signal indicating the formation of a thicker layer. HeLa cells showed adhesive properties on both surfaces, however, greater biosensor signal was obtained on cell adhesion to gelatin at 0.2 mg/ml (see Fig. 4c,d).

Taken together, the application of 0.2 mg/ml gelatin solution resulted in a more homogenous and thinner layer allowing the deposition of larger cell masses inside the evanescent field. Due to these favorable characteristics, we employed a 0.2 mg/ml gelatin coating in all of the further experiments with HUVECs.

### The Epic BT-based EnLaB system is an optimal platform for the label-free investigation of complex EC responses to plasma serine protease treatment

**Figure 5:**
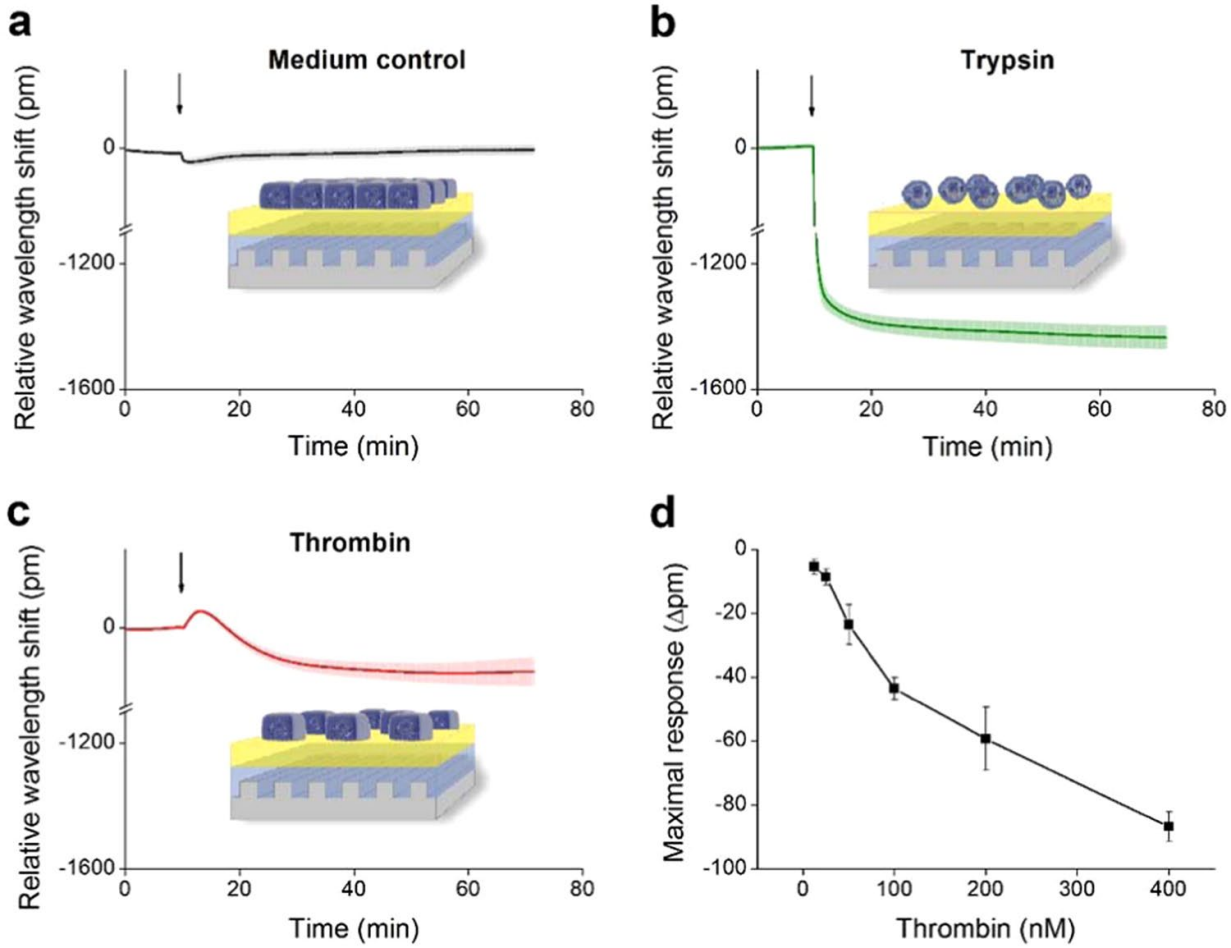
Fine-tuning of the label-free measurement of dynamic mass redistribution on confluent HUVEC monolayers. A 384 well biosensor microplate was precoated with 0.2 mg/ml gelatin (37 °C, 20 min, PBS), then wells were washed with PBS and confluent monolayers of HUVECs were created on the surface (24 h). The microplate was placed into the Epic BT reader, and after the stabilization of the baseline, cell treatments were added directly to the culture medium, and the wavelength shift was monitored for 60 min. (**a**) The culture medium was added alone. (**b**) 2 μM of trypsin was added. (**c**) 300 nM of thrombin was added. (**d**) Various concentrations of thrombin were added. The response curves were normalized to the medium control and the maximum values of the wavelength shift were determined. The figures show representative graphs of at least 3 independent measurements.

The Epic BT seemed to be an ideal candidate platform for the label-free investigation of complex EC behavior. We have successfully created stable, confluent endothelial monolayers on the 0.2 mg/ml gelatin coated biosensor surface of 384-well plates. Adding growth medium to EC monolayers did not induce any changes in the wavelength shift curve (Fig. 5a). On the contrary, the total detachment of ECs from the surface in response to trypsin treatment resulted in a great negative wavelength shift of −1400 pm (Fig. 5b). However, thrombin – a serine protease known to trigger the contraction of ECs and formation of paracellular gaps, thereby reversibly increasing endothelial permeability – induced a more moderate, but still definite negative wavelength shift (Fig. 5c). The effect of thrombin was further investigated in 6 different concentrations in the range of 12.5 nM up to 400 nM, and was found to be dose-dependent (p < 0.001, ANOVA, post test for linear trend, Fig. 5d). According to these results, EnLaB appeared to be suitable for testing the effects of plasma serine proteases on ECs.

### New hits identified using EnLaB – plasma serine proteases kallikrein, C1r and MASP-2 induced characteristic wavelength shift curves

**Figure 6:**
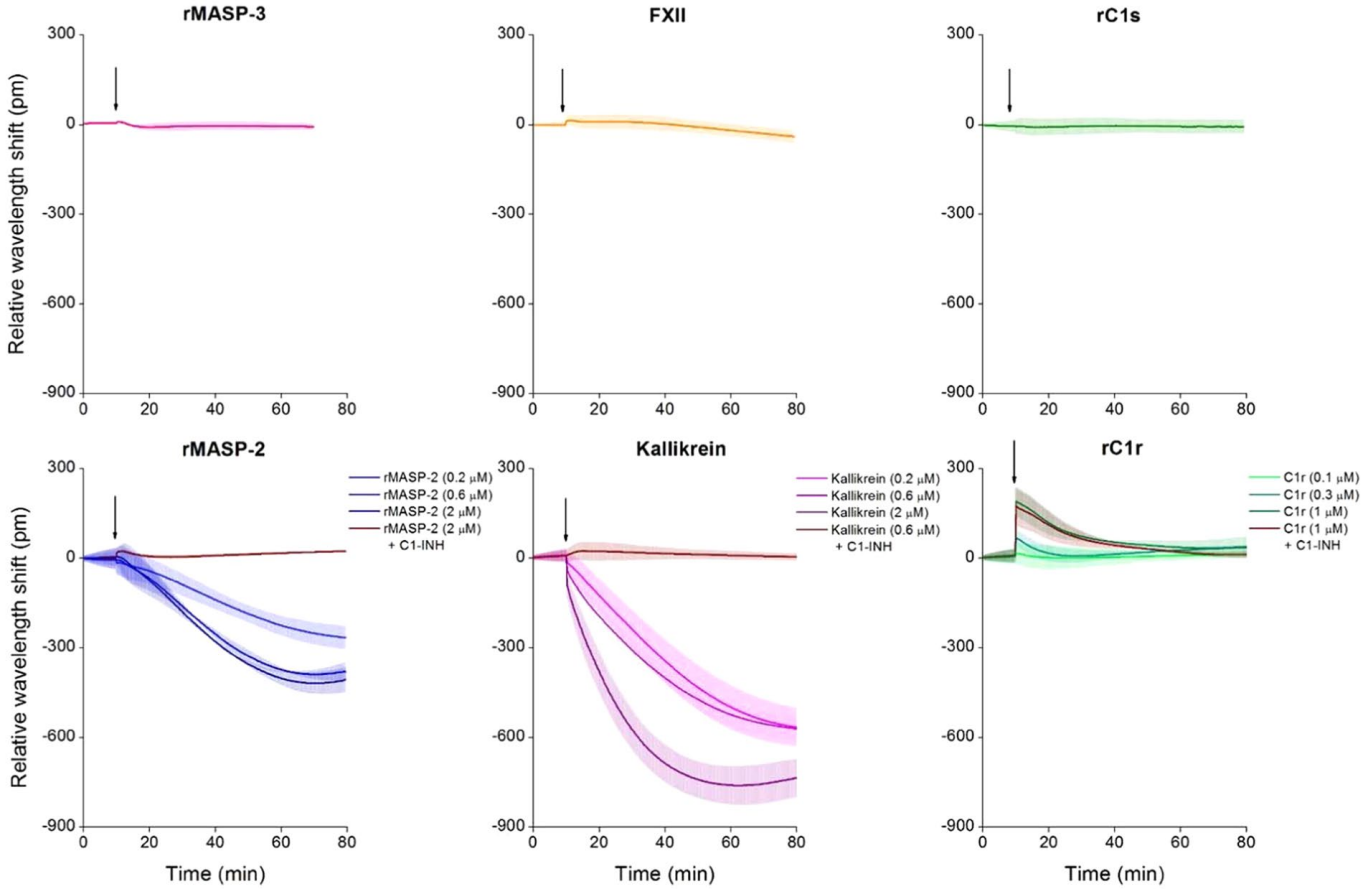
Effects of plasma serine proteases on the dynamic mass redistribution of confluent endothelial monolayers. A 384 well biosensor microplate was precoated with 0.2 mg/ml gelatin (37 °C, 20 min, PBS), then the wells were washed with PBS, and confluent monolayers of HUVECs were grown on the surface (24 h). The microplate was placed into the Epic BT reader, and after the stabilization of the baseline, serine proteases rMASP-3 (1 μM), FXII (2 μM), rC1s (1 μM), rMASP-2 (0.2, 0.6 or 2 μM) and kallikrein (0.2, 0.6 or 2 μM), rC1r (0.1, 0.3 or 1 μM) or culture medium alone was added. Proteases rMASP-2, kallikrein and rC1r were also applied in complex with their inhibitor, C1-INH (using a three-fold molar excess of C1-INH). Cell treatments were added directly to the culture medium, and the wavelength shift was monitored for 60 min. Wavelength shift curves were normalized to the medium control. The figures show representative graphs of at least 3 independent measurements.

After fine-tuning the system, we screened active plasma serine proteases that have not yet been investigated thoroughly on endothelial monolayers, such as rMASP-2, rMASP-3, kallikrein, rC1s, rC1r and FXII. From the tested enzymes, rMASP-3, rC1s, and rFXII did not alter EC behavior, but rMASP-2, rC1r and kallikrein treatment resulted in characteristic wavelength shift curves significantly different from the medium control (Fig. 6). Proteases rMASP-2 and kallikrein both induced a negative wavelength shift, while rC1r had an opposite effect, resulting in a positive signal. The effect was dose-dependent in all three cases.

Preincubating rMASP-2 and kallikrein with their natural inhibitor, C1-inhibitor (C1-INH) - which results in covalent complex formation - abolished the effects of these proteases. Interestingly, C1-INH was ineffective in the case of rC1r, although this protease also forms enzymatically inactive covalent complexes with C1-INH assessed by PAGE (data not shown).

### Validation of the new hits in well-established assays - rMASP-2 and kallikrein triggered intracellular Ca^2+^ mobilization, while all three proteases increased endothelial permeability by the disruption of adherent junctions

**Figure 7:**
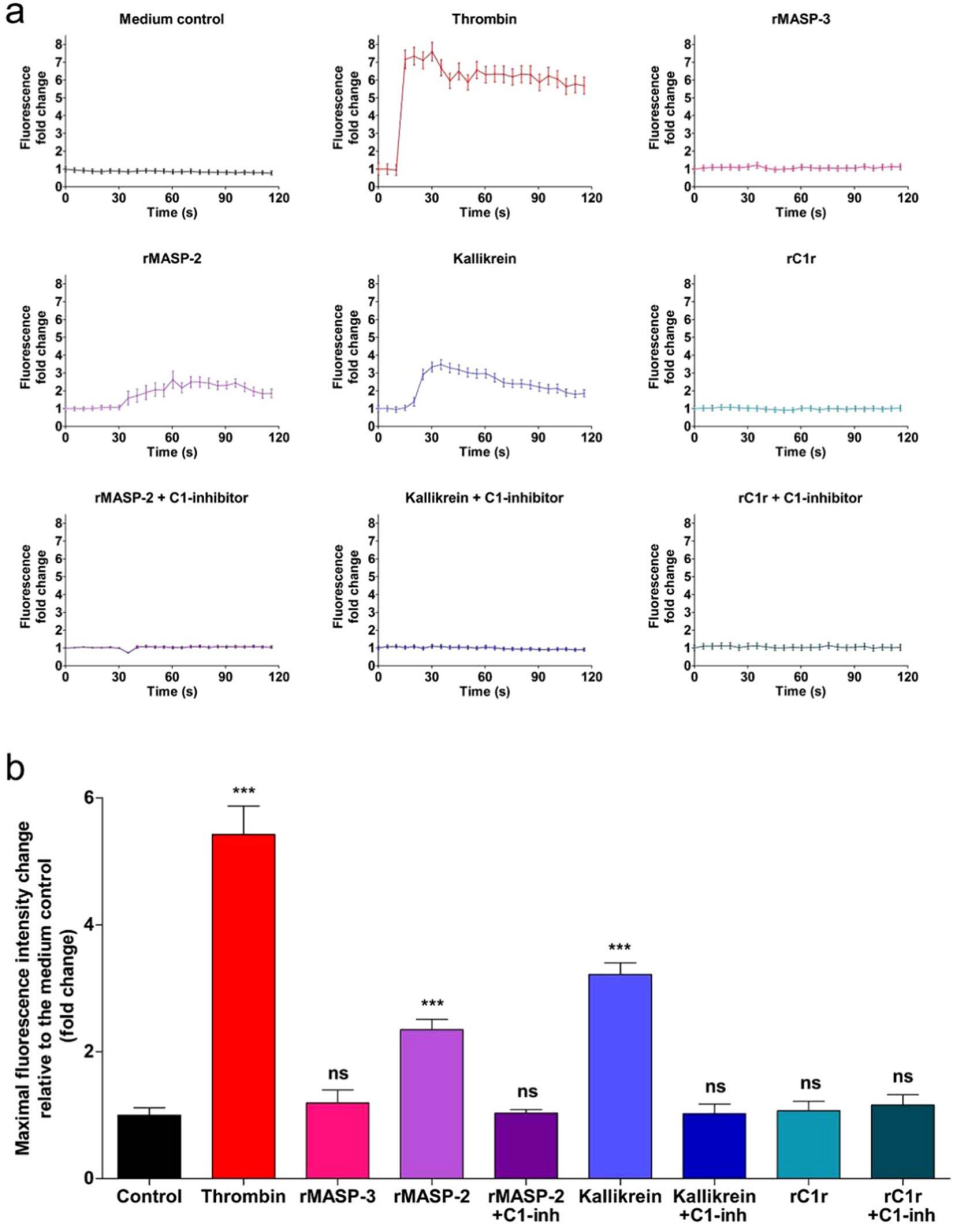
Effects of plasma serine proteases on intracellular Ca^2+^ mobilization of HUVECs. Confluent layers of HUVECs were seeded onto 96 well microplates and cultured for 24 h. Cells were loaded with 2 μM Fluo-4-AM for 20 min, then incubated in HBSS for another 20 min. Sequential images were obtained every 5 s by fluorescence microscopy. Three photos were taken to determine the baseline fluorescence, then cells were treated with thrombin (300 nM), rMASP-3 (1 μM), rMASP-2 (2 μM), rMASP-2/C1-INH complex (2 μM/ 6 μM), kallikrein (2 μM), kallikrein/C1-INH complex (2 μM/ 6 μM), rC1r (1 μM), rC1r/C1-INH complex (1 μM/ 3 μM), or culture medium alone and the response was monitored for 2 min. Twenty cells per image were analyzed using the CellP software (Olympus). (**a**) Graphs from single, representative experiments. Fluorescence fold change values relative to medium control is shown, except in case of Medium control, where all kinetic values are normalized to the first fluorescence value. (**b**) Means of the maximum fluorescence intensity values of 3 independent experiments, normalized to the control.

To validate our results obtained using EnLaB, we first performed an intracellular Ca^2+^ mobilization assay. Treating HUVECs with rMASP-2 and kallikrein induced a Ca^2+^ response similar to thrombin used as positive control. The response was completely blocked by C1-INH in the case of both rMASP-2 and kallikrein. On the other hand, rC1r could not elicit a Ca^2+^ response. rMASP-3 was used to represent those proteases that did not alter the wavelength shift curve in the Epic BT system, and consistently with those results, it failed to trigger a Ca^2+^ signal (Fig. 7).

**Figure 8:**
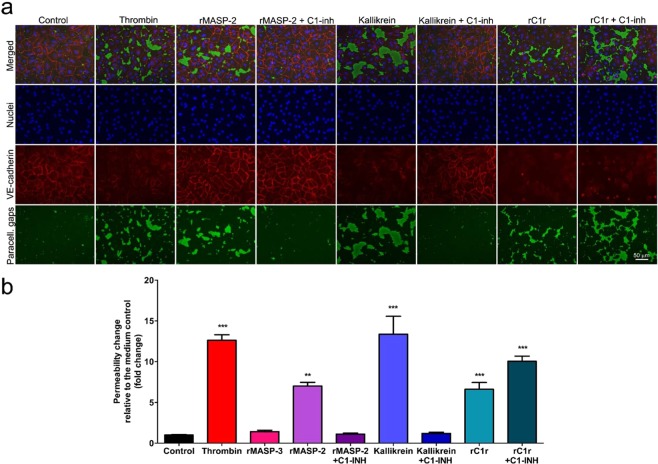
Effects of plasma serine proteases on VE-cadherin adhesion junctions and the permeability of HUVEC monolayers. (**a**) Permeability tests were carried out using a modified version of the XPerT technique. Briefly, confluent layers of HUVECs were seeded onto biotinylated gelatin-precoated 96-well microplates and cultured for 2 days. Cells were treated with thrombin (300 nM), rMASP-3 (1 μM), rMASP-2 (2 μM), rMASP-2/C1-INH complex (2 μM/ 6 μM), kallikrein (2 μM), kallikrein/C1-INH complex (2 μM/ 6 μM), rC1r (1 μM), rC1r/C1-INH complex (1 μM/ 3 μM), or culture medium alone for 20 min. Streptavidin-Alexa488 was added to each well for 2 min and cells were fixed with 2% paraformaldehyde-PBS. VE-cadherin adhesion molecules were stained with anti-VE-cadherin antibody followed by an Alexa 568-conjugated seconder antibody. Cell nuclei were stained using Hoechst 33258. (**b**) The total area of the streptavidin-Alexa 488 stained paracellular gaps was determined on each image using the CellP software. Fold-change data are the mean (+SEM) of three independent experiments calculated after normalization to the medium control. **p < 0,001; ***p < 0,0001, relative to the control (One-way ANOVA with Tukey post-test).

Then, we assessed whether rMASP-2, kallikrein or rC1r can affect endothelial permeability, similarly to thrombin. According to our results, all three proteases can disrupt EC-EC adhesion by triggering the disappearance of VE-cadherin from the AJs (Fig. 8a), which resulted in a significant permeability increase, 7-fold in the case of rMASP-2, 13.4-fold for kallikrein and 6.6-fold for rC1r (Fig. 8b).

In the case of rMASP-2 and kallikrein, the effects could also be blocked by C1-INH. However, – in accordance with the results obtained using the Epic BT system – the rC1r/C1-INH complex induced an even greater monolayer permeability (10-fold) than rC1r alone (Fig. 8b). Interestingly, treating HUVECs with rC1r or the rC1r/C1-INH complex resulted in the total, homogenous loss of VE-cadherin – in contrast with the other proteases tested, which caused the disappearance of VE-cadherin only in the close proximity of the paracellular gaps (Fig. 8a). The plasma derived full-length form of C1r – pC1r – also induced the loss of VE-cadherin, increased endothelial permeability and – similarly to rC1r –, it could not be blocked by C1-INH, the pC1r/C1-INH complex induced even stronger responses (data not shown).

## Discussion

Here we report the successful development of a new, label-free assay suitable for the real-time monitoring of complex endothelial cell behavior. Using this method, we are the first to describe novel functions – directly related to endothelial cell activation – for three important plasma serine proteases.

There is an increasing demand for label-free technologies and an expanding number of available experimental systems that enable us to monitor cell behavior without the perturbation of cell signaling. Therefore, we aimed to develop an assay that takes advantage of these new opportunities. In the newly developed EnLaB assay, we used the Epic BT platform – a resonant waveguide grating-based technique, which is outstanding among other label-free technologies as it combines the possibility of high-throughput screening with the ability of recording complex cellular responses coming from both intracellular and extracellular events. Although the impedance-based xCELLigence system is also capable of the label-free monitoring of cell cultures in a high-throughput screening mode^29^, it is not suitable for the detection of complex cell behavior, as impedance changes result mostly from the changes in cell adhesion and not intracellular rearrangements. On the other hand, other label-free techniques – such as SPR – are usually incapable of high-throughput screening of cellular effects.

It has to be also emphasized that the optimization of surface coatings in evanescent field-based biosensor applications is a crucial step, since the surface coating connects the biological layer (in our case, the endothelial) with the optical transducer. Layer thickness, hydration degree and functionality need to be carefully considered. In cell-based applications, the layer has to be thin compared to the penetration depth of the evanescent wave, but still has to provide full coverage and biological functionality. In the present work, the combined analysis of data obtained from parallel optical and mechanical biosensor measurements proved to be an attractive tool to determine both the hydration and viscoelastic properties of hydrated nanolayers deposited onto the surface of the biochips. Using EnLaB, we investigated confluent EC monolayers on gelatin precoated 384-well microplates mounted by optical biosensors. We studied the thickness and roughness of the gelatin-coated surface, which was intended to mimic the function of the basal membrane naturally supporting the cells from beneath. A gelatin coating applied in a concentration of 0.2 mg/ml was found to be optimal for our purposes. OWLS, QCM and AFM measurements showed that this concentration creates a homogenous and highly hydrated layer thin enough for the biosensor to “see through”, while demonstrating ideal shear viscosity and shear elastic modulus values for the proper mechanical support of ECs. As the confluent HUVEC monolayer is a widely accepted *in vitro* model of the inner vessel wall, we successfully created a homogenous, confluent monolayer of HUVECs on the gelatin-coated biosensor surface.

After the fine-tuning of cell culturing, a simple and quick protocol for biosensor experiments was worked out using serine proteases – e.g. thrombin and trypsin – that are known to affect EC monolayer properties. As our new assay appeared to be an effective method for the label-free screening of various substances on ECs, in the current paper we investigated the possible EC-related effects of a subset of plasma serine proteases yet underinvestigated on ECs. From the serine proteases tested, MASP-2, C1r and kallikrein induced characteristic signals fundamentally different from the untreated control. These proteases were further tested on ECs using well-established assays and their EC-related functions were verified.

MASP-2 and C1r are key serine proteases of the complement cascade – the most important humoral arm of innate immunity, which provides an immediate response against pathogens entering our blood and other bodily fluids.

MASP-2 plays a central role in the lectin pathway of the complement system, which can be activated by the sugar motifs of various bacteria and fungi. The importance of MASP-2 is underlined by the fact that in its absence, lectin pathway ceases without the formation of the C3 convertase complex^30,31^, which is a prerequisite for the effector functions of the complement system. To date, no direct cellular effects have been described for MASP-2, although Asgari *et al*. showed that MASP-2 is critically important in the pathomechanism of renal ischemia–reperfusion injury independent of its substrate, complement C4^32^. In our new label-free assay, treating HUVECs with rMASP-2 resulted in a characteristic response curve. This effect was found to be dose-dependent and could be blocked by C1-INH, the natural inhibitor of many plasma serine proteases (also including C1r and kallikrein). We further investigated the effects of rMASP-2 on EC monolayers in traditional assays. The results of these tests were in accordance with the biosensor experiments; rMASP-2 was able to induce intracellular Ca^2+^ mobilization in HUVECs, which is a classical sign of G-protein coupled receptor (GPCR) mediated EC activation. Moreover, rMASP-2 induced a marked disintegration of VE-cadherin – one of the most important adhesion molecules of endothelial intercellular adhesion junctions –, and significantly increased endothelial permeability. As all of these effects were blocked by the serine protease inhibitor C1-INH, it is plausible that similarly to MASP-1^17^ and thrombin^33,34^, the protease activity of MASP-2 is required for its cellular effects and suggest that the receptor of MASP-2 is among protease-activated receptors (PARs).

C1r is essential for the initiation of the classical pathway of the complement system, which can be activated by IgM or IgG containing immunocomplexes. The absence of C1r is known to contribute to the development of the severe autoimmune condition systemic lupus erythematosus (SLE)^35^, although no direct cellular effects have yet been linked to this protease. However, we found that the rC1r treatment of HUVEC monolayers caused a characteristic wavelength shift curve in the Epic BT system. The effect was dose-dependent, but, surprisingly, could not be blocked by C1-INH, which is the only known inhibitor of C1r.

Moreover, unlike all the other proteases assessed, rC1r induced a positive wavelength shift in Epic BT system, similarly to histamine (data not shown). Although wavelength shift is proportional to the mass redistribution in the evanescent field, the exact molecular mechanisms behind the effect is largely unknown. On the other hand, rC1r and also the C1-INH/rC1r complex failed to elicit an intracellular Ca^2+^ mobilization in HUVECs (which was the same in the case of pC1r). However, rC1r (and also pC1r) treatment of HUVECs resulted in a dramatic, homogenous loss of the adhesion molecule VE-cadherin, which was even more pronounced in the case of C1r/C1-INH complexes. Consequently, C1r (both recombinant and plasma derived forms) significantly increased endothelial permeability, and this response was also greater when ECs were treated with C1r/C1-INH complexes. Taken together, these observations suggest that unlike thrombin, MASP-1 or MASP-2, C1r is suspected to affect EC monolayer properties independent of its protease activity (as C1-INH could not block the effects of C1r). This leads to the assumption that the EC receptor of C1r is not among PARs.

Kallikrein is the central protease of the kinin-kallikrein system and the main contributor to the generation of the proinflammatory mediator bradykinin, a small vasoactive peptide with a wide variety of physiological/pathological roles (e.g. edematogenic, vasodilatory, angiogenic and hyperalgesic effect)^36,37^. The results of Buillet *et al*. suggested that kallikrein is also able to directly increase endothelial permeability independent of bradykinin^38^. The authors linked this effect to the kallikrein-mediated cleavage of VE-cadherin on ECs, but the phenomenon was not sufficiently investigated. Consistent with these results, we found that kallikrein may indeed affect EC monolayer properties as it caused a characteristic wavelength shift curve. This effect was dose-dependent and could be completely blocked by C1-INH. In addition, we showed that kallikrein is able to induce intracellular Ca^2+^ mobilization in HUVECs, suggesting that it can also activate intracellular signal transduction pathways in ECs. Moreover, kallikrein treatment caused the disruption of VE-cadherin adhesion junctions of HUVECs and significantly increased endothelial permeability. Both effects were blocked by C1-INH. Reconciliation of our results and the findings of Buillet *et al*. suggests that kallikrein has a triple effect on EC monolayers. Besides its contribution to the generation of the edematogenic factor bradykinin, kallikrein can directly affect endothelial permeability – not only by the cleavage of adhesion molecule VE-cadherin, but also by the activation of ECs, which can lead to an active contraction of the cells. As the latter effect of kallikrein was also likely to require its protease activity (it was blocked by C1-INH), the EC receptors of kallikrein are also possibly among PARs.

Our findings that plasma serine proteases MASP-2, C1r, and kallikrein can affect EC behavior are of high physiological relevance, as these proteases are in direct contact with the endothelium. Due to their permeability-increasing effect, there is a possibility that MASP-2, C1r and kallikrein can play a role in the pathomechanism of life-threatening circulatory conditions – e.g. hereditary angioedema or sepsis – where edema formation and activation of plasma protease cascades occur simultaneously.

The challenges we faced during this study outline the directions for further research. One such direction is to increase the duration of experiments with EnLab. Although, in this phase of the study we aimed to investigate the short-term effects of the proteases (as the signaling of PARs typically manifests in an early cellular response), we are planning to develop an incubator compatible arrangement with sample handling, which would allow long-term monitoring of cellular monolayers and their responses to external stimuli. Another future plan is to develop the method to be able to distinguish between different species in the same sample. It is quite clear from the literature as well as from our own experiments that the answer of the cells to the stimulus applied is rather complex. At this point, we envision EnLab primarily as a screening platform to find EC activating factors, but if a database of such cellular signals is built up, spectral decomposition might predict the main acting species in the sample. An additional exciting question is to investigate the mechanism of action of C1r. The fact that the cellular effects of rC1r could not be blocked with C1- inhibitor – which otherwise inhibited its enzymatic activity – suggests that rC1r does not exert its effect through its protease activity, cleaving endothelial PARs. Probably, rC1r activates its receptor through a receptor-ligand binding interaction, possibly further stabilized by C1-inhibitor. This issue would be rather important to clarify, since no C1r-receptor has been revealed so far.

The most important strength of our method is that it offers a simple and label-free alternative for the high-throughput screening of various agents on ECs and other epithelial cells, therefore we plan to broaden the spectrum of the molecules and cell types to be investigated with EnLab. Our search for additional EC activating plasma proteases will continue as well as we intend to study the effects of several other molecules, even possible drug candidates. As we consider the EnLaB assay suitable for studying monolayers of all adherent cell types, we also plan to adapt the assay for other endothelial and epithelial cell types. Our goal is to model different tissues, where a cell monolayer is exposed to various types of agonists or is especially important in transport processes (e.g. models of the intestinal villi, or glomerular epithelial cells).

In conclusion, our newly developed EnLaB assay was found to be suitable for the identification of agents with a potential of affecting EC monolayer properties. The advantageous properties of EnLaB makes it an ideal platform for both basic research to assess cellular functions of adherent cells and also for pharmaceutics to test libraries of drug candidates.

## Methods

### Reagents

Due to the limited accessibility of high-purity plasma derived forms, we used recombinant catalytic fragments of human MASP-1, MASP-2, MASP-3, C1s and C1r, all of them containing only the CCP1, CCP2 and SP domains of the proteases (hereinafter referred to as rMASPs, rC1s and rC1r). The recombinant proteases were produced in *E. coli*^39^ and purified as described earlier^40^. The preparations were found to be free of bacterial contaminations (routinely checked as described earlier). Furthermore, it was shown in our previous study that the plasma purified, full-length form of rMASP-1 is functionally equivalent to the recombinant catalytic fragment as the two forms elicit the same cellular responses^41^.

In the case of thrombin, FXII and kallikrein, human plasma derived forms were used, while trypsin was produced from bovine plasma. Effects of rC1r were validated with human plasma derived C1r (pC1r).

Other reagents were purchased from Sigma-Aldrich, unless stated otherwise.

### Preparation and culturing of human umbilical vein endothelial cells (HUVECs)

HUVECs were isolated by collagenase digestion from fresh umbilical cords obtained during normal deliveries of healthy neonates as described earlier by our group^42,43^. Cells were cultured in gelatin-precoated flasks (Corning® Costar®) in MCDB-131 medium (Life Technologies), completed with 5% heat-inactivated fetal calf serum (FCS), 2 ng/ml human recombinant epidermal growth factor (hrEGF, R&D Systems), 1 ng/ml human recombinant basic fibroblast growth factor (hrBFGF), 0.3% Insulin Transferrin Selenium (Life Technologies), 1% Chemically Defined Lipid Concentrate (Life Technologies), 1% Glutamax (Life Technologies), 1% Penicillin-Streptomycin antibiotics, 5 μg/ml Ascorbic acid, 250 nM Hydrocortisone, 10 mM HEPES, and 7.5 U/ml Heparin (hereinafter referred to as Comp-MCDB). Cells were cultured in 5 mg/ml gelatin precoated cell culture flasks (Corning) until use.

For permeability tests and measurements of intracellular Ca^2+^ mobilization, a modified version of AIM-V medium (Life Technologies) was used, completed with 1% filtrated, heat inactivated bovine serum (PAN Biotech), 1 ng/ml hrBFGF growth factor, 2 ng/ml hrEGF (R&D Systems), and 7.5 U/ml Heparin (hereinafter: Comp-AIM-V).

HUVECs were used from passages 2 to 4, and each experiment was repeated with cells from two to four different individuals. This study was conducted in accordance with the WMA Declaration of Helsinki, and the protocol was approved by the Semmelweis University Institutional Review Board (permission number: TUKEB141/2015). Written informed consent was provided by all participants prior to inclusion.

### HeLa cells culture

HeLa cells (Sigma-Aldrich) were maintained in Dulbecco’s modified Eagle’s medium, supplemented with 10% fetal bovine serum (Biowest SAS, France), 4 mM L-glutamine, 100 U/ml penicillin and 100 µg/ml streptomycin solution. Cells were cultured in a humidified atmosphere containing 5% CO_2_ at 37 °C. On reaching 80% confluence, cells were detached every 3–4 days using 0.05% (w/v) trypsin, 0.02% (w/v) EDTA solution.

### Optical waveguide lightmode spectroscopy

The sensing principle of OWLS is based on the evanescent waves of the guided waveguide modes^18,19,21^. For the measurements, a BIOS-1 instrument (MicroVacuum Ltd., Hungary) was used with planar optical waveguide, which consists of a SiO_2_-TiO_2_ sol-gel waveguide layer with grating on a glass substrate (OWLS chip type 2400 μV, MicroVacuum Ltd., Hungary). The grating serves to in-couple the He-Ne laser beam (632.8 nm) into the chip, and zeroth-order transverse electric (TE) and transverse magnetic (TM) modes are excited in the waveguide. The chips were equipped in a microfluidic assembly mounted with a septum. A peristaltic pump (Ismatec, Reglo) maintained the constant 1 μl/s flow in the liquid cell^44^. The refractive indices of the different solutions (n_c_) were measured by a refractometer at 632.8 nm (J157 Automatic refractometer, Rudolph). To evaluate the recorded OWLS data, a 4-layer mode equation was applied^45^. The experiments were carried out at room temperature. The steps of the experiments were the following: stable PBS baseline, 20 min 0.2 mg/ml gelatin flow, washing with PBS until stable signal, 20 min 5 mg/ml gelatin flow, washing with PBS until stable signal, again 20 min 5 mg/ml gelatin flow, washing with PBS until stable signal.

### Quartz crystal microbalance

The QCM technique measures the frequency shift in the resonance of a piezoelectric quartz crystal, generated by an added mass on the sensor surface. At the same time, the dissipated energy of the oscillation is also recorded, enabling to characterize the viscoelastic behavior of the deposited adlayer. The *in situ* QCM measurements were performed by a QCM-I (QCM with impedance measurement) instrument (MicroVacuum Ltd., Hungary) using sensor chips with resonance frequency of 5 MHz^22^. The gold surface of the chips was covered by the same SiO_2_-TiO_2_ sol-gel waveguide material that is applied in OWLS. The chips were mounted in a microfluidic assembly equipped with a septum. The constant 1 μl/s flow rate was maintained by a peristaltic pump. The QCM experiments were carried out in parallel to the OWLS experiments in the same environmental conditions and performing the same experimental steps (see the above description for the OWLS measurements). The measured fundamental and overtone frequencies were the following: 5, 15, 25, 25 MHz, corresponding to the overtone numbers of *n* = 1, 3, 5, 7.

The obtained frequency and dissipation shift data (*Δf*_*n*_, *ΔD*_*n*_) were evaluated and the viscoelastic analysis was performed by our developed MATLAB code applying Voinova’s Voigt-based viscoelastic model^25^.

### Atomic force microscopy

AFM was performed with a commercial instrument (Nanosurf FlexAFM, Nanosurf, Switzerland). All imaging was performed under liquids (PBS). The images were recorded in dynamic mode with a Multi75Gd-G type probe. The recorded images were evaluated by the Gwyddion software.

### Epic BT biosensor technique

The Epic BT instrument (Corning Incorporated, Corning, NY, USA) illuminates a 96- or 384-well microplate (#5080, #5040, Corning Incorporated, Corning, NY, USA) – mounted by an individual RWG-based sensor in each well – and noninvasively monitors refractive index changes in a ~150 nm deep evanescent electromagnetic field over the sensor surface. An RWG-based optical biosensor consists of a waveguiding layer of Nb_2_O_5_ supported by a corrugated glass substrate. The characteristic wavelength, at which the waveguide mode and evanescent field is created, is called the resonant wavelength. Cellular responses upon stimuli change the local refractive index inside the evanescent field, thus the resonant wavelength shifts to a new value and the biosensor signal is recorded kinetically in real time. The instrument scans the wavelength between 825 and 840 nm in every 3 s each well of the microplate allowing 96 or 384 parallel measurements^3,6,46^.

### Preparation of the Epic biosensor surface with gelatin coatings

For the Epic BT measurement, gelatin was applied in two concentrations (0.2 and 5 mg/ml in PBS) to provide a biocompatible surface. The sensor surface coatings were created by adding 60 μl of gelatin solutions to the wells of 96-well Epic cell assay microplate with further incubation for 30 min at 37 °C. There are several ways to prepare gelatin coating on a solid support. Thereafter, to compare the usually applied protocol steps for coating preparations and its subsequent influence on layer properties, the following steps were performed in three different ways: i) no further manipulation was applied, ii) gelatin solution was aspirated and PBS was added to the wells, iii) wells of the biosensor plate were rinsed two times with PBS.

### HeLa cell adhesion assay on gelatin-coatings

Upon surface modification by gelatin-coatings and Epic BT measurement, all wells were rinsed with 20 mM HEPES HBSS pH 7.4 (assay buffer) three times, and baselines were recorded in the wells with 60 μl of assay buffer for 1 h. Meanwhile, HeLa cells were removed from tissue culture dishes using a trypsin-EDTA solution. Trypsin digestion was terminated by the addition of completed medium and the harvested cells were centrifuged at 200 × g for 5 min. The cell pellet was resuspended in assay buffer and 20 μl of cell suspension containing 20,000 cells were added to the sensor wells and signals were recorded for additional 130 min. All measurements were repeated at room temperature three times.

### Label-free measurement of dynamic mass redistribution on confluent endothelial monolayers (EnLaB)

384-well biosensor microplates were coated with 0.2 mg/ml gelatin – diluted in PBS – at 37 °C for 20 min and then wells were washed with PBS2 times. HUVECs were seeded onto the coated surface of biosensor microplate and were let attach in a humidified incubator for a confluent monolayer formation for 24 h.

An Epic biosensor plate with adhered cells was let cool down to room temperature for approximately 15 min before starting the experiment to prevent condensation, and then was placed into an Epic BT reader. Stable baselines had been established for all wells, compounds from the source plate were added to the wells using an electronic 16-channel pipette Finnpipette™ Novus (Thermo Fisher Scientific, Waltham, MA, USA) in stepping mode. Cells were treated with plasma serine proteases trypsin (2 μM), thrombin (used as a positive control in every experiment in 300 nM, Merck-Millipore), FXII (2 μM, Innovative Research), kallikrein (0.2, 0.6 and 2 μM, Innovative Research), rMASP-2 (0.2, 0.6 and 2 μM), rMASP-3 (1 μM), rC1s (1 μM), rC1r (0.1, 0.3 and 1 μM), pC1r (1 μM, Merck-Millipore). Proteases rMASP-2, kallikrein and rC1r were also applied in complex with human plasma derived C1-INH (Berinert®, CSL Behring, added in 3 times the molar amount of the proteases and incubated together for 30 min before cell treatment). Complex formation was checked by polyacrylamide gel electrophoresis. Protease solutions were directly added to the growth medium and wavelength shift changes were monitored for 60 min after cell treatment. Wavelength shift curves were normalized by subtracting the values of the medium control (negative control). All treatments were repeated within each experiment at room temperature three times, and experiments were repeated using ECs from different individuals.

### Permeability measurements and visualization of VE-cadherin adhesion molecules

We used a modified version of the recently developed XPerT technique^47^ for permeability measurements. Briefly, confluent layers of HUVECs were seeded onto 96 well plates precoated with 0.25 mg/ml biotinylated gelatin and cultured in Comp-AIM-V for 2 days. Following cell treatment, Streptavidin-Alexa488 (2 µg/ml, Life Technologies) was added to each well for 2 min. After cell fixation (1% paraformaldehyde-PBS) 2 pictures were taken of each well with an Olympus IX-81 fluorescence microscope mounted by an Olympus XM-10 camera. To determine the size of the stained area, quantitative image analysis was carried out on each image using the CellP software (Olympus). Thrombin (300 nM) was used as a positive control in every experiment.

To allow covalent complex formation, human plasma derived C1-INH (the natural inhibitor of MASP-2 and C1r) was preincubated with the proteases in a 3-fold molar excess for 30 min before treating HUVECs.

Adhesion molecule VE-cadherin was stained with anti-VE-cadherin (Santa Cruz) antibody, followed by Alexa568 labeled goat-anti-rabbit secondary antibody. Nuclei of the cells were labeled with Hoechst 33258 (200 ng/ml, Molecular Probes).

### Intracellular Ca^2+^ mobilization assay

Mobilization of intracellular Ca^2+^was measured as described by our group earlier^48^. Briefly, confluent layers of HUVECs were seeded onto 96 well plates and cultured in Comp-AIM-V for 1 day. Cells were loaded with 2 µM Fluo-4-AM (Molecular Probes) for 20 min, then incubated in HBSS for another 20 min. Sequential images were obtained every 5 seconds by fluorescence microscopy. Baseline fluorescence was determined from the first three images, then cells were treated with the serine proteases or complexes of these proteases with C1-INH (added in 3-fold molar excess) and incubated together for 30 min before the treatment of the cells). The response was recorded for 2 min. Fluorescence intensity change of twenty cells was analyzed on each image with the CellP software.

### Statistical analysis

All experiments were performed in triplicates and repeated using HUVECs from at least two different individuals. The statistical analysis was carried out using GraphPad Prism version 5.01 (GraphPad software, La Jolla, California, USA). To determine statistical significance of the results, one-way ANOVA with Tukey post test was conducted, where a p value of ≤0.05 was considered significant.
